# 3D Digital Anatomic Angioarchitecture of the Rat Spinal Cord: A Synchrotron Radiation Micro-CT Study

**DOI:** 10.3389/fnana.2020.00041

**Published:** 2020-07-22

**Authors:** Ping Li, Yan Xu, Yong Cao, Tianding Wu

**Affiliations:** ^1^Department of Obstetrics, Xiangya Hospital, Central South University, Changsha, China; ^2^Hunan Engineering Research Center of Early Life Development and Disease Prevention, Changsha, China; ^3^Department of Sports Medicine, Xiangya Hospital, Central South University, Changsha, China; ^4^Key Laboratory of Organ Injury, Aging and Regenerative Medicine of Hunan Province, Changsha, China; ^5^Department of Spine Surgery and Orthopaedics, Xiangya Hospital, Central South University, Changsha, China; ^6^National Clinical Research Center for Geriatric Disorders, Xiangya Hospital, Central South University, Changsha, China

**Keywords:** spinal cord microvasculature, SRμCT, digital anatomy, virtual micro-endoscopy, 3D print

## Abstract

Comprehensive analysis of 3D angioarchitecture within the intact rat spinal cord remains technically challenging due to its sophisticated anatomical properties. In this study, we aim to present a framework for ultrahigh-resolution digitalized mapping of the normal rat spinal cord angioarchitecture and to determine the physiological parameters using synchrotron radiation micro-CT (SRμCT). Male SD rats were used in this *ex vivo* study. After a proportional mixture of contrast agents perfusion, the intact spinal cord covered the cervical spinal from the upper of the 1st cervical vertebra to the 5th lumbar vertebra was harvested and cut into proper lengths within three distinct regions: Cervical 3–5 levels, Thoracic 10–12 levels, Lumbar 3–5 levels spinal cord and examined using SRμCT. This method enabled the replication of the complicated microvasculature network of the normal rat spinal cord at the ultrahigh-resolution level, allowing for the precise quantitative analysis of the vascular morphological difference among cervical, thoracic and lumbar spinal cord in a 3D manner. Apart from a series of delicate 3D digital anatomical maps of the rat spinal cord angioarchitecture ranging from the cervical and thoracic to the lumbar spinal cord were presented, the 3D reconstruction data of SRμCT made the 3D printing of the spinal cord targeted selected microvasculature reality, that possibly provided deep insight into the nature and role of spinal cord intricate angioarchitecture. Our data proposed a new approach to outline systematic visual and quantitative evaluations on the 3D arrangement of the entire hierarchical microvasculature of the normal rat spinal cord at ultrahigh resolution. The technique may have great potential and become useful for future research on the poorly understood nature and function of the neurovascular interaction, particularly to investigate their pathology changes in various models of neurovascular disease.

## Introduction

A wide range of spinal cord-related neurovascular diseases and other pathological processes are associated with vascular alterations (Wu et al., 2008; Heldner et al., 2012). Such as the cavernous hemangiomas in the spinal cord consist of abnormal blood vessels with poor blood flow (Pilz et al., 2020). The spinal dural arteriovenous (AV) fistulas are the most commonly encountered vascular malformation of the spinal cord and still underdiagnosed entities (Takai et al., 2020). A comprehensive exploration of the entire 3D arrangement and structural parameters of the spinal cord microvasculature under normal physiological conditions is undoubtedly the prerequisite to monitor vascular changes during pathological processes. The angioarchitecture of the spinal cord has unique 3D characteristics and is more complicated than that of any other known biological organization (Morishita et al., 2003; Mazensky et al., 2011; Santillan et al., 2012). Establishing advanced imaging methods for proper 3D visualization of the spinal cord vasculature network at ultrahigh resolution has been raised great concern (Prestigiacomo et al., 2003; Dray et al., 2009; Hu et al., 2014). However, there is a lack of a complete map of the entire microvasculature of the rat spinal cord and are thus limited for further understanding its function.

The synchrotron light source is electromagnetic radiation produced by the acceleration of electrons that move near the speed of light through magnetic fields (Suortti and Thomlinson, 2003; Qu et al., 2015). The advent of synchrotron radiation micro-CT (SRμCT) has emerged as a promising *ex vivo* technology that provides a high brilliant radiation light source that is suitable for ultrahigh-resolution imaging of the 3D microstructure of biological specimen (Heinzer et al., 2008; Connor et al., 2009; Lu et al., 2012). Regarding the detection of the vasculature in the central nervous system, SRμCT imaging has been broadly applied in the rat brain (Stolz et al., 2011; Lu et al., 2012; Schrötter et al., 2013). However, seldom focused on the extremely intricate and delicate nature of the spinal cord microvasculature.

In the current study, we applied the ultrahigh-resolution SRμCT imaging to visualize the 3D angioarchitecture of the rat spinal cord. A systematically comparative study and 3D quantitative analysis were conducted to demonstrate that this method is a powerful tool for assessing vascular architecture in ultrahigh resolution. Additionally, Furthermore, the data obtained from SRμCT could serve as a template for a specialized, custom-built 3D printer to manufacture a scaffold that mimics the exact geometry of the 3D spinal cord microvasculature, which could provide a new approach for (spinal cord injury) SCI regeneration repair. Our data proposed an approach that outlined the systematic visual and quantitative evaluations of the hierarchical vasculature of the rat spinal cord, which could serve as a new platform for the pre-clinical *ex-vivo* investigation of neurovascular networks.

## Materials and Methods

### Animals

A total of eight adult male SD rats (250–350 g body weight) were used for the study which were obtained from the Animal Center of Central South University, Changsha, and kept in a temperature-controlled room and had free access to food and water. Animal care and use were performed following the guidelines established by the Animal Care and Use Committee. The protocol was approved by the Committee on the Ethics of Animal Experiments of Central South University (No. 201704033).

### Preparation of Imaging Specimens

Specimens were prepared as previously described with some modifications (Hu et al., 2014). After the rats were deeply anesthetized, a thoracotomy was rapidly performed to expose the heart. Heparinized saline was rapidly infused into the circulatory system *via* the ascending aorta, allowing an effective drain of blood flow. Then, 10% buffered formalin was perfused trans-cardially for tissue fixation. A proportional mixture of contrast agents (Microfil MV-122, Flow-Tech, CA, USA), described previously (Hu et al., 2014), was infused into the ascending aorta *via* a perfusion pump. The whole length of the spinal cord was harvested and post-fixed in a 10% buffered formalin solution. The spinal cord specimens were rinsed and then dehydrated with a gradient of ethyl alcohol. The specimens were dried in the air before SRμCT scanning.

### SRμCT Scanning

The intact rat spinal cord specimens were cut into proper lengths within three regions: Cervical 3–5 levels enlargement of the spinal cord, Thoracic 10–12 levels, Lumbar 3–5 levels enlargement of the spinal cord. Different regions were scanned using SRμCT performed at the BL13W1 beamline of the Shanghai Synchrotron Radiation Facility (SSRF, China; Hu et al., 2014). The monochromatic X-ray energy was adjusted to 15 keV, the exposure time was set to 2.5 s, and the sample-to-detector distance (SDD) was adjusted to 2 cm. The minimal spatial resolution of the SRμCT technique is 3.25 μm. After SRμCT scanning, all initial projection images were transformed into 2D slice sections using the PITRE Reconstruction software (applied *via* the BL13W1 experimental station) based on the filtered back-projection (FBP) algorithm. A series of 2D slices were reconstructed using Image Pro Analyser 3D software (Version 7.0, Media Cybernetics, Inc., USA) to generate the 3D images.

### 3D Vascular Quantitative Analysis

**Figure 1:**
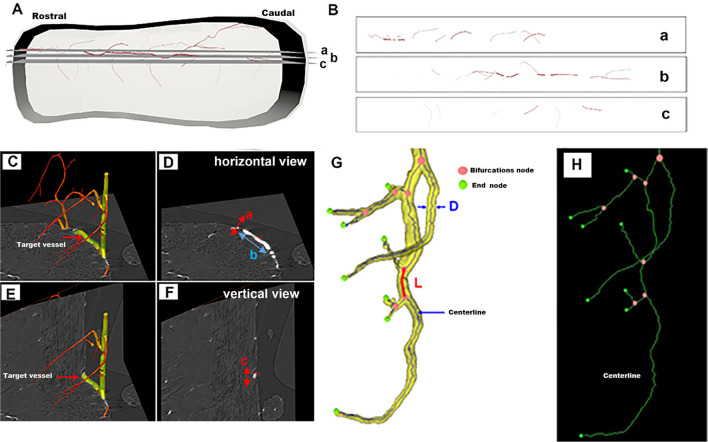
Schematic depiction of a randomly selected vessel within the intact spinal cord for 3D quantitative measurement. **(A)** The 3D view of the one typical vessel within the intact spinal cord. **(B)** The corresponding 2D virtual section selected from panel **(A)**. **(C–F)** The vessel measurement from horizontal and vertical views. **(G)** Vessel tree displaying the parameters used for the 3D quantification of the networks of vessel branches characterized by their length *L*, diameter *D*, and bifurcation points. **(H)** The centerline skeleton of the vessel tree.

Figure 1A shows a schematic depiction of one typical vessel within the intact spinal cord. The selected corresponding 2D virtual tissue sections are shown in Figure 1B. In the 2D view, a, b, and c correspond to multiple distinct vessels. They arise from the same vessel from a 3D view as shown in Figure 1A. As listed in Figure 1D, the 2D diameter measurement of randomly chosen vessels marked in Figure 1C from the horizontal view was incorrect due to the lack of complete 3D visualization. However, the diameter calculated from the vertical view of the specific vessel (Figures 1E,F) was accurate and could reflect the authenticity of the vessel diameter.

Thus, to objectively analyze the vascular network quantitatively, the 3D vessel tree skeleton model for vascular structure characterization was generated (Risser et al., 2009; Hintermuller et al., 2010; Figure 1G). After the vasculature was segmented from the spinal cord parenchyma with Image Pro Analyser 3D software (Version 7.0, Media Cybernetics Inc., USA) based on the iterative gray-level threshold algorithm (Reichold et al., 2009), we performed the following protocol for the calculation of the 3D vascular morphological parameters (Kim et al., 2008). (1) Vectorization, skeletonization based on the voxel erosion (Lang et al., 2012) plugin in Image Pro Analyser 3D software, was used to automatically extract the centerline of the vessel tree (Figure 1H). (2) Once the centerline was extracted, numerous vascular quantification parameters such as vessel segment numbers, vessel segment density, vessel segment length, etc could be systematically analyzed and robustly quantified for complex vessel structure. Figure 1G defined the schema of vessel tree parameters. A vessel segment was defined as a section of vessels between two bifurcation points. The vessel length L, labeled red in Figure 1G, was measured by the sum of the distances between the two bifurcation points. A 3D Euclidean distance map (EDM) of the vasculature described previously was evaluated using the Chamfer Map module plugin in Image Pro Analyser 3D software to determine the vessel radius (Ghanavati et al., 2014). The diameter of vessel thickness was calculated from the vessel radius Rm, the mean radius Rm leading to the mean diameter *D* = 2 Rm. The vessel volume fraction represented the ratio between the number of voxels belonging to the vasculature and the total number of voxels in the spinal cord.

### 3D Vascular Printing

After the 3D angioarchitecture of the spinal cord was segmented by the ImagePro Analyzer 3D software, the ROI including the anterior spinal arteries (ASA), and the central sulcus artery (CSA), were extracted from the SRμCT data using surface rendering and converted to STL (STereoLithography; surface data as an aggregation of triangular meshes) format. The final STL files were loaded in the ZEditTM3.21 software program (3D systems, USA) to adjust the x, y, and z coordinates of the model to the desired printer size (800× magnification), which allowed for proper visual inspection. Then, they were imported into the 3D printer (Objet 350 Connex, Stratasys, USA) to produce the final 3D printer model of the spinal cord microvasculature. VeroMagenta was selected to print the vascular structures. A comparative analysis was performed to evaluate the accuracy of the 3D printing model with the SRμCT findings.

### Statistical Analysis

Data are presented as the mean ± standard deviation (SD). Data analysis was performed with SPSS 21.0 (SPSS Inc., Chicago, IL, USA). The data were analyzed with a one-way analysis of variance (ANOVA) followed by Tukey’s method for the *post hoc* test to observe the changes among different groups on the morphological parameters. *p* < 0.05 was considered to be significant.

## Results

### Characterization of the 3D Angioarchitecture Morphology Among Three Spinal Cord Regions

**Figure 2:**
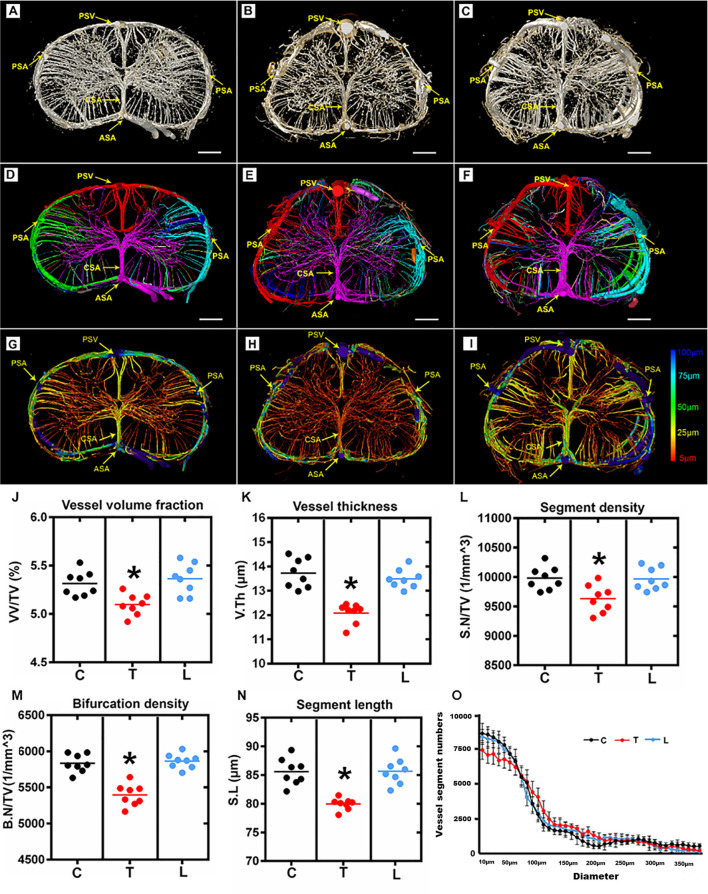
Overview and quantification of the 3D morphology of the rat spinal cord microvasculature. **(A–C)** The raw image of the spinal cord microvasculature among cervical, thoracic, and lumbar regions. **(D–F)** Representative images of the segmented vasculature and the color-coded labeled vessels of the spinal cord among the three regions. The major arteries and veins including the posterior spinal artery (PSA), central sulcus artery (CSA), anterior spinal artery (ASA), and the posterior spinal vein (PSV). **(G–I)** The pseudocolored image of the spinal cord microvasculature among three regions. The color bar on the left of the panel indicates the associated vessel diameters. The smallest capillaries are depicted in red and the largest vessels in blue. **(J–N)** The mean vessel volume fraction, vessel thickness, segment density, bifurcation density, and segment length were relatively lower in the thoracic region than the cervical and lumbar spinal cord; *p < 0.05. **(O)** Vessel distribution histogram revealed the vessel thickness in the thoracic region with a diameter of less than 25 μm was slightly lower than that of the cervical and lumbar regions. C, cervical spinal cord; T, thoracic spinal cord; L, lumbar spinal cord. *p < 0.05. Scale bar = 200 μm.

The entire rat spinal cord microvasculature was visualized by SRμCT. We obtained highly detailed spinal cord 3D digitalized angioarchitecture maps of the cervical, thoracic, and lumbar spinal cord regions. As presented in Figure 2, one anterior spinal artery (ASA), two posterolateral spinal arteries (PSA), and one posterior spinal vein (PSV) were observed along the surface of the reconstructed 3D images, which gave a distinct depiction of the complete spatial spinal cord microvascular network. From the vertical view, the general outline of the microvasculature among three spinal cord regions varied, but the intrinsic artery network had the same constitution and could be separated into a central sulcal arterial system and a peripheral arterial system. The central sulcal artery (CSA) originated from the ventral ASA and branched into the bilateral gray matter at the anterior white commissure. The PSA gave off many branches that coursed slightly rostral before entering the posterior gray matter of the spinal cord. Additionally, in the vertical view, the gray matter where the penetrating branches of the CSA and that of the peripheral artery transversely and longitudinally anastomose into a rich microvascular network was characterized by a unique butterfly shape.

The corresponding 3D vascular images with local features of each vascular segment were automatically color labeled following segmentation based on the connectivity of spinal cord microvascular branches (Figures 2D–F). Furthermore, the pseudo-color image of the spinal cord microvasculature that depicted the vessel diameters was presented in Figures 2G–I.

The vascular morphometry analysis revealed lower vessel volume fraction in the thoracic spinal cord region compared with the cervical and lumbar spinal cord (Figure 2J). The investigation of vessel thickness indicated a smaller vessel diameter in the thoracic spinal cord region (Figure 2K). Additionally, the vascular segment density, bifurcation density, and segment length measured in the thoracic region were similarly lower than the cervical and lumbar spinal cord (Figures 2L–N). Furthermore, the distribution of vessel thickness among the three spinal cord regions was evaluated. Interestingly, we found the vessel thickness in the thoracic region with a diameter of less than 25 μm was slightly lower than the cervical and lumbar regions, but not for vessels with larger diameters (Figure 2O). There were also no differences between the cervical and lumbar regions detected in the distribution of vessel thickness.

### Characterization of the 3D Morphology of Central Sulcus Arteries Among the Three Spinal Cord Regions

**Figure 3:**
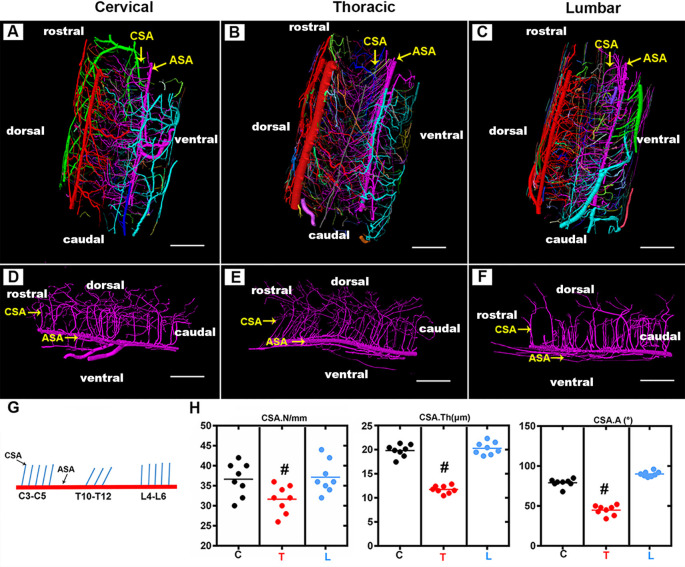
Overview of the 3D morphology of the rat spinal cord central sulcus arteries among the cervical, thoracic, and lumbar regions. **(A–C)** The lateral view of the 3D morphology of the intact rat spinal cord microvasculature. **(D–F)** The sagittal view of the 3D morphology of the central sulcus arteries of the cervical, thoracic, and lumbar spinal cord. **(G)** The schematic depiction of the morphology of the central sulcus arteries among the cervical and thoracic lumbar spinal cord regions. **(H)** The quantitative data of the central sulcus arteries among three regions. the mean number (/mm) of CSA in the thoracic region was lower than that of the cervical and lumbar regions, ^#^*p* < 0.05. The vessel thickness (μm) of the CSA in the thoracic region was smaller than that in the cervical and lumbar regions, ^#^*p* < 0.05. No major difference was found between the cervical and lumbar regions regarding these two morphological parameters. The average angle (°) between CSA and ASA in the thoracic region was acute and presented the smallest value among the three regions, ^#^*p* < 0.05. C, cervical spinal cord; T, thoracic spinal cord; L, lumbar spinal cord. ASA, anterior spinal arteries; CSA, central sulcus arteries. N, the mean number; Th, the vessel thickness; A, the average angle. Scale bar = 200 μm; ^#^*p* < 0.05.

The CSA, which supplies two-thirds of the nutritional demands of the spinal cord, plays a key role in maintaining the normal function of the spinal cord. As shown in the 3D digital angioarchitecture map of the spinal cord in Figure 3, the CSA usually exhibited a narrow diameter at the origin site before entering the neural parenchyma. It became inosculated with the peripheral arteries and knitted into a spatial microvasculature network that feeds the neural parenchyma of the gray matter. The ASA was located on the ventral surface of the spinal cord, while, the large PSV was located on the dorsal surface of the spinal cord. We also found that the CSA and peripheral arteries were terminal branches and had no precapillary interconnections (Figures 3A–C).

**Figure 4:**
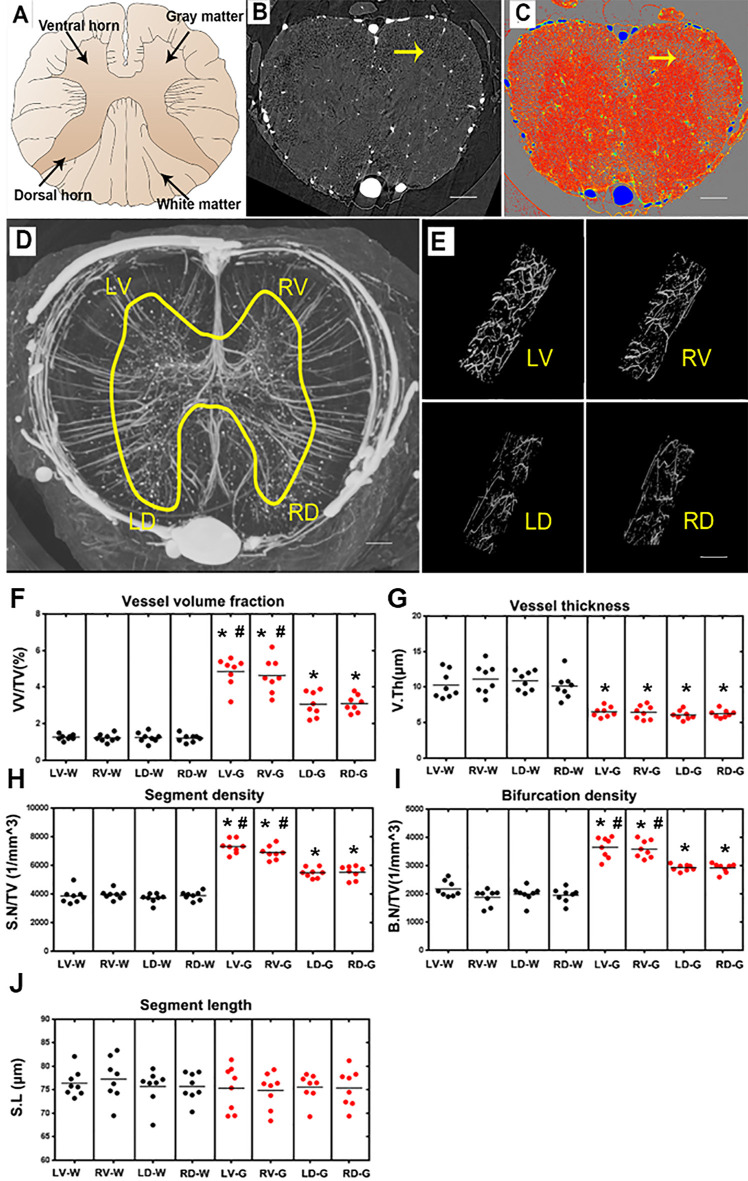
Characterization of the morphology of the normal blood supply of the thoracic spinal cord and quantification of the morphological changes between gray and white matter in the LV, RV, LD and RD horn. **(A)** Schematic depiction of the spinal cord anatomy. **(B)** A randomly selected synchrotron radiation micro-CT (SRμCT) slice. **(C)** The pseudocolor slice image of SRμCT. Arrow in **(B,C)** indicates the boundary between gray and white matter. **(D)** 3D image of the spinal cord microvasculature. **(E)** 3D morphology of the microvasculature randomly selected from four gray matter regions of the spinal cord. **(F)** The vessel volume fraction was mainly elevated in gray matter structures. **(G)** Vessel thickness displayed a contrary tendency with low vessel thickness in gray matter structures, *p* < 0.05. The distribution of vessel thickness revealed a leftward shift in gray matter, *P* < 0.05. **(H)** Segment density was marginally higher in gray matter structures, *p* < 0.05. **(I)** The higher bifurcation density in gray matter was correlated with the increased segment density, *p* < 0.05. **(J)** Segment length showed no difference between gray and white matter. LV, left ventral; RV, right ventral; LD, left dorsal; RD, Right dorsal; LV-G, left ventral gray; RV-G, right ventral gray; LD-G, left dorsal gray; RD-G, right dorsal gray; LV-W, left ventral white; RV-W, right ventral white; LD-W, left dorsal white; RD-W, right dorsal white. **(B–D)** Scale bar = 200 μm. **(E)** Scale bar = 20 μm. *Significantly different from the gray matter and white matter at (*p* < 0.05). ^#^Significantly different from the ventral gray and dorsal gray (*p* < 0.05).

By providing a clear depiction of the main spatial vascular arrangement of the CSA, we found that the CSA formed a distinct angle with the axis of the ASA with a variable range from the cervical to the thoracic and lumbar spinal cord (Figures 3D–F). The schematic depiction of the morphology of the CSA was drawn in Figure 4G, the mean number of CSA per millimeter of spinal cord ASA in the thoracic region was lower than that of the cervical and lumbar regions (Figure 3H). The vessel thickness of the CSA in the thoracic region was also smaller than that in the cervical and lumbar regions (Figure 3H). No major difference was found between the cervical and lumbar regions regarding these two morphological parameters. The average angle between CSA and ASA in the thoracic region was acute and presented the smallest value among the three regions (Figures 3H,G). However, the CSA in the cervical and lumbar regions showed straight or right-angle configurations with the ASA before entering the anterior median sulcus of the spinal cord parenchyma (Figures 3H,G).

### Characterization of the 3D Angioarchitecture of Thoracic Spinal Cord

A systematic qualitative and quantitative evaluation of the thoracic spinal cord angioarchitecture is displayed in Figure 4. A unique butterfly shape was present in the gray matter in the raw slice image of the SRμCT and consisted of the schematic graph (Figures 4A,B). We observed a boundary line located at the junctional zone of the gray and the white matter of the spinal cord, which was obvious in the pseudocolor slice image marked with a yellow arrow in Figure 4C. The 3D angioarchitecture image of the thoracic spinal cord listed in Figure 4D and was divided into four main areas (left and right dorsal region and left and right ventral region) within each area. Representative 3D images of the areas of interest of the microvasculature in the gray matter of the four main areas were randomly selected and demonstrated in Figure 4E.

After quantitative analysis, increased vessel volume fraction, segments, and bifurcation density were found in all investigated gray matter structures (LV-G: left ventral gray; RV-G: right ventral gray; LD-G: left dorsal gray; RD-G: right dorsal gray; Figures 4F,H,I). In particular, the value among these parameters was slightly higher in the ventral horn (LV-G and RV-G) than in the dorsal horn structure (LD-G and RD-G; Figures 4F,H,I). However, the vessel thickness displayed a contrary tendency characterized by a smaller distribution of vessel thickness in the gray matter than white matter (Figure 4G). Interestingly, we also found the segment length revealed no differences between the gray and white matter structure, despite the higher vessel volume fraction, segment, and bifurcation density in gray matter structures (Figure 4J). These results suggested that an extraordinary number of intrinsic vessel branches with small diameters within the gray matter of spinal cord neuro parenchyma.

### 3D Printing of Thoracic Spinal Cord Microvasculature

**Figure 5:**
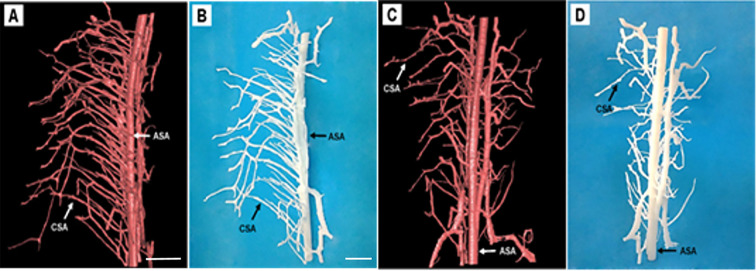
Representative 3D printed model of the rat spinal cord microvasculature. **(A,C)** The spinal cord vasculature template obtained from SRμCT for different perspectives. **(B,D)** The corresponding 3D printed spinal cord vasculature model shows the complex interweaving of vessel branches. ASA, anterior spinal arteries; CSA, central sulcus arteries. **(A,C)** Scale bar = 200 μm; **(B,D)** scale bar = 5 cm.

One current strategy for studying the microstructure is to virtually print the specimen structure where each vessel morphology could be built into reality. To make the major vessel morphology obtained from SRμCT more clearly, we removed most of the vessel branches, leaving the two main vessel branches, including the CSA and the ASA within the specimen (Figures 5A,C). After printable models of the thoracic spinal cord microvasculature are built, they can be magnified and printed at a proper size 800× larger than the actual size, which makes the smallest CSA noticeable. This application is helpful to construct vascular scaffolds with high biological structure simulation to repair spinal cord injuries in the field of tissue engineering (Figures 5B,D).

## Discussion

In recent years, there has been growing interest in the studying of 3D spatial properties of the microvasculature in the central nervous system, particularly the neurovascular diseases that have been strongly associated with vascular alterations (Heinzer et al., 2006; Vasquez et al., 2011; Zhang et al., 2015). Thus the thorough knowledge of the anatomical architecture of the spinal cord under normal physiological status is undoubtedly a pivotal foundation and could provide reliable biological information for the study of variations in the spinal cord microvasculature (Heinzer et al., 2006).

In the current study, we present a 3D digital anatomic atlas of the angioarchitecture of the healthy rat spinal cord using high-resolution SRμCT imaging (Heinzer et al., 2008). Additionally, we computed the average diameter of the vessel. Different colors represented the vessel diameter throughout the entire spinal cord microvasculature system. It was thus possible to visualize the changes in vessel diameter and vessel arrangement at a glance throughout the entire spinal cord. The 3D morphology of the ASA, CSA, PSA, PSV could be vividly visualized. The ASA travels along the anterior sulcus of the spinal cord. The ASA and PSA constitute longitudinal arterial plexuses. The main blood supply to spinal gray matter is derived from CSA, which arises from the ASA. These main arteries form a complex network that provides blood supply to maintain the normal function of the spinal cord.

The quantification of vessel density and shape parameters has traditionally been performed using 2D histologic techniques (Figley et al., 2014). However, relatively few quantitative descriptive parameters in the 3D morphology of the microvasculature in the rat spinal cord are available (Fratini et al., 2015; Yao et al., 2015). Indeed, 3D measurements are more accurate than those quantitative parameters derived from 2D images. Some crucial data, including the vessel length and trajectories, will be extremely difficult to measure on 2D tissue sections. In this study, a series of parameters that directly describe the 3D spinal vasculature arrangement was calculated. Compared to the microvasculature in the cervical and lumbar region, the vessel branches were relatively lower in the thoracic region and were consistent with those previously reported (Williams et al., 1991; Biglioli et al., 2004). The CSA at the thoracic region emerged at an acute angle before penetrating the parenchyma and had the narrowest vessel diameter among the three regions of the spinal cord. This may explain the difference in the hemorheology of the spinal cord among various regions. The cervical and lumbar regions are supplied by the large CSA, whereas the thoracic region has a small central supply (Biglioli et al., 2004; Martirosyan et al., 2011). Thus, the obstruction of the artery feeding the cervical and lumbar regions seldom results in infarction. Due to the poor blood supply in the thoracic spinal cord region, the compromise of local blood flow creates a great risk of ischemia and is harmful to the thoracic spinal cord region (Jacobs et al., 2002; Backes et al., 2008; Melissano et al., 2010). Previous studies have shown an approximately 4% incidence of spinal cord infarction following the repair of aneurysms in the descending thoracic aorta (Svensson, 1996; Melissano and Chiesa, 2009; Martirosyan et al., 2011). Our study provides valuable biological imaging evidence to elucidate the underlying mechanism of the vascular hemodynamics of the spinal cord in the thoracic region.

The 3D printing technique artificially generates specimens’ morphologies in 3D and offers a new form of data visualization (Muth et al., 2014; Kiefer et al., 2015; McDougal and Shepherd, 2015). Designing a vascular graft of small diameter with comparable mechanical and biological properties to living tissues remains challenging (Zhang et al., 2017). The specific microvascular structures data with high precision obtained from SRμCT can be transformed into a physically realizable object through a 3D printing technique, which offers a new approach for obtaining transplanted grafts that mimic the biomechanical properties of native blood vessels (McClure et al., 2010). The 3D visualize microvasculature by SRμCT offers a new tool for us to digital mapping the morphological parameter of a complex specimen at high resolution.

Although the SRμCT technique has remarkable superiority for spinal cord vessel visualization, it still possesses some limitations. The anatomical features of the spinal cord make the implementation of *in vivo* imaging in the study of organs in living rat a challenging task due to the anatomy features that near the lungs and heart, which generates significant movement artifact, causing the *in vivo* imaging of the spinal cord microvascular failed. Additionally, as scanning requires a long time, high-resolution imaging usually implies a high radiation dose (Suortti and Thomlinson, 2003; Lu et al., 2012), which doubtlessly delivers radiation damage to the living specimen scanning. The application of synchrotron radiation (SR) has been applied to visualize the cerebral blood vessel *in vivo* after the contrast perfusion. It means that SRμCT will be a promising tool for the *in vivo* visualization of the vessel once the above mention problems are resolved.

## Conclusion

In conclusion, our study proposed a new approach that provided the systematic visual and quantitative evaluations on the hierarchical microvasculature at an ultrahigh-resolution based on the SRμCT technique, which offers substantial biological information on the anatomical features of the vascular network in the rat spinal cord. The present method has great potential and becomes a useful tool for future research on the poorly understood nature and function of the neurovascular interaction, particularly to investigate their pathology changes in various models of neurovascular disease.

## Data Availability Statement

All datasets generated for this study are included in the article.

## Ethics Statement

The animal study was reviewed and approved by the Committee on the Ethics of Animal Experiments of Central South University.

## Author Contributions

PL, YX, and YC carried out experiments, analyzed the data, and contributed to writing the original draft. YC and TW designed the study, analyzed the data and reviewed/edited the manuscript. All authors have read and approved the final version of the manuscript.

## Conflict of Interest

The authors declare that the research was conducted in the absence of any commercial or financial relationships that could be construed as a potential conflict of interest.
